# Клинический случай гипопитуитаризма вследствие гипофизита в периоде реконвалесценции после перенесенной инфекции COVID-19

**DOI:** 10.14341/probl12896

**Published:** 2022-03-24

**Authors:** Н. Ю. Горбова, В. П. Владимирова, Л. Я. Рожинская, Ж. Е. Белая

**Affiliations:** Национальный медицинский исследовательский центр эндокринологии; Национальный медицинский исследовательский центр эндокринологии; Национальный медицинский исследовательский центр эндокринологии; Национальный медицинский исследовательский центр эндокринологии

**Keywords:** новая коронавирусная инфекция, COVID-19, гипофизит, гипофиз, магнитно-резонансная томография

## Abstract

**ЦЕЛЬ:**

ЦЕЛЬ. Представление первого клинического случая частично обратимого гипопитуитаризма у пациентки с подтвержденным гипофизитом после перенесенной инфекции СOVID-19.

**МАТЕРИАЛЫ И МЕТОДЫ:**

МАТЕРИАЛЫ И МЕТОДЫ. У пациентки с клиническими проявлениями гипопитуитаризма после перенесенного СOVID-19 были проанализированы клиническая картина, лабораторные показатели и данные МРТ в динамике.

**РЕЗУЛЬТАТЫ:**

РЕЗУЛЬТАТЫ. У пациентки 35 лет развились клинические симптомы гипопитуитаризма через 2 мес после подтвержденной инфекции COVID-19. Лабораторное обследование подтвердило пангипопитуитаризм, на МРТ — признаки гипофизита. Через 4 мес симптомы стали менее выраженными и появились признаки восстановления по лабораторному обследованию: уровни кортизола сыворотки крови, адренокортикотропного гормона, пролактина, тиреотропного гормона, свободных тироксина и трийодтиронина — в норме. Однако гипогонадизм и гипокортицизм сохранялись. По данным МРТ проявления гипофизита уменьшились. Полное восстановление гипофизарно-гонадной и гипофизарно-тиреоидной оси зарегистрировано в октябре 2021 г. с восстановлением менструального цикла, но сохраняется вторичный гипокортицизм.

**ВЫВОДЫ:**

ВЫВОДЫ. Приводятся доказательства отсроченного поражения гипофиза после заражения вирусом COVID-19 с частичным восстановлением его функции и структуры. На данный момент механизмы воздействия не совсем понятны, необходим дальнейший сбор данных.

## Abstract (en)

**AIM:**

AIM. To present a clinical case of reversible hypopituitarism due to hypophysitis developed after COVID-19 infection.

**MATERIALS AND METHODS:**

MATERIALS AND METHODS. A patient with residual clinical manifestations of hypopituitarism underwent clinical evaluation at the time of symptoms of hypopituitarism and in follow-up. Morning serum cortisol (171–536 nmol/l) was measured by electrochemiluminescence immunoassay. Morning ACTH (7.2–63.3 pg/ml), prolactin (66–436 mU/l), TSH (0.25–3.5 mIU/L), fT4 (9–19 pmol/l) and fT3 (2.6–5.7 pmol/l) were measured by chemiluminescence immunoassay. Data were analyzed throughout the course of the disease.

**RESULTS:**

RESULTS. A 35-year-old female developed clinical symptoms of hypopituitarism two months after recovery from a confirmed COVID-19 infection. Laboratory investigation confirmed hypocorticism, hypothyroidism, hypogonadism and the patient was prescribed appropriate hormonal therapy in January 2021. Four months later the symptoms were alleviated (April 2021) and there were signs of recovery shown by imaging and hormonal: morning serum cortisol 227 nmol/l, morning ACTH 33.96 pg/ml, prolactin 68.3 mU/l, TSH 2.626 mIU/L, fT4 10.75 pmol/l, fT3 3.96 pmol/l. Thyroid hormone was discontinued, but hypogonadism and hypocorticism persisted with estradiol — 51.48 pmol/l, 24h urine cortisol level — 41.8 nmol/day. MRI results showed that the signs of hypophysitis were alleviated in comparison with MRI from January 2021. Full recovery of pituitary axis was reported in October 2021, with recovery of normal menstrual cycle. Furthermore, hormonal profile was likewise normal.

**CONCLUSION:**

CONCLUSION. This report provides evidence of delayed damage to the pituitary gland after infection with the COVID-19, with recovery of its function and structure. To date, the mechanisms of such an impact are not entirely clear; further collection of data on such cases and analysis is required.

## АКТУАЛЬНОСТЬ

Новая коронавирусная инфекция 2019 (COVID-19) значительным образом влияет на социальную жизнь, бросает вызовы системам здравоохранения по всему миру. По мере того, как накапливаются данные, становится очевидно, что осложнения могут коснуться любых органов и систем, в том числе и эндокринной. В первом масштабном российском исследовании 3480 пациентов, больных COVID-19, 13,6% имели диабет 2 типа, который значимо повышал риск летального исхода [1].

Европейское эндокринологическое общество указывает на большее количество осложнений COVID-19 в группе больных сахарным диабетом и делает акцент на тщательном контроле гликемического профиля для снижения риска заражения [2]. Также в литературе рассматривается ведение пациентов с ожирением, надпочечниковой недостаточностью, болезнью Иценко–Кушинга, несахарным диабетом, остеопорозом [3–7]. Однако исследований отдаленного влияния COVID-19 на гипоталамо-гипофизарную систему по-прежнему мало. По этой причине мы описываем уникальный случай пациентки с впервые возникшим гипофизитом и, как следствие, пангипопитуитаризмом в периоде реконвалесценции после перенесенной инфекции COVID-19.

## Представление клинического случая

Пациентка А., 35 лет, с начальными проявлениями рассеянного склероза (по данным медицинской документации) и перенесенной коронавирусной инфекцией в анамнезе поступила в отделение нейроэндокринологии и остеопатий ФГБУ «НМИЦ эндокринологии» МЗ РФ (ЭНЦ), 19.04.2021 с жалобами на отсутствие менструаций в течение 4 мес, снижение оволосения на лобке и подмышечных впадинах, периодически общую и мышечную слабость, «дрожание мышц».

С 2018 г. пациентку беспокоят головные боли — при МРТ головного мозга заподозрили рассеянный склероз. Наблюдается у невропатолога, лечение не назначали. Регулярно выполняла МРТ головного мозга из-за предполагаемого рассеянного склероза, и в августе 2020 г. при очередном исследовании обнаружили кисту кармана Ратке (рис. 1).

В октябре-ноябре 2020 г. перенесла COVID-19-инфекцию в легкой форме (потеря обоняния, вкуса, субфебрильная температура), подтвержденную положительным ПЦР-тестом. Через 2 мес впервые отметила отсутствие менструаций, снижение оволосения в подмышечных впадинах, на лобке, нарастание слабости, присоединились тошнота, головокружения, потеря веса, спазмы мышц, ломота в суставах.

При лабораторном обследовании по месту жительства на момент пика жалоб (январь 2021) выявлены признаки пангипопитуитаризма: вторичный гипокортицизм, вторичный гипотиреоз, вторичный гипогонадизм, гиперпролактинемия, дефицит соматотропного гормона: клинические и лабораторные признаки несахарного диабета отсутствовали (табл. 1).

Лечение было назначено спустя месяц от появления первых признаков гипопитуитаризма — пациентка получала заместительную терапию гидрокортизоном 20 мг в сутки, левотироксином 50 мкг ежедневно натощак и каберголином 1 мг в неделю. Через несколько недель от начала приема гидрокортизона отметила появление избыточной энергии, повышенной активности, в связи с этим самостоятельно снизила дневную дозу гидрокортизона до 5 мг, иногда вовсе пропускала дневную зону (принимала на 10 мг гидрокортизона утром). Самостоятельно отменила прием левотироксина в апреле 2021 г., длительность терапии составила 2 мес. Приведенные данные лабораторных анализов от апреля 2021 г. — на фоне отмены левотироксина, в дальнейшем данный препарат не принимала. На МРТ через 2 мес после перенесенной коронавирусной инфекции обнаружены увеличение ранее выявленной кисты кармана Ратке, а также утолщение воронки гипофиза, признаки инфундибулогипофизита (см. рис. 1).

При стационарном исследовании в ФГБУ «НМИЦ эндокринологии» (апрель 2021 г.) клинических признаков гипокортицизма и гипотиреоза выявлено не было, что подтверждали результаты лабораторного исследования (табл. 1). Что касается вторичного гипогонадизма, уровни лютеинизирующего (ЛГ) и фолликулостимулирующего (ФСГ) гормонов стали нормальными (4,73 и 4,52 соответственно), однако отмечалось значимое снижение эстрадиола — 51,48 пмоль/л (97–592). При этом масса тела оставалась нормальной — 52 кг при росте 166 см, ИМТ 18,9 кг/м2. На фоне отмены гидрокортизона выявлено снижение свободного кортизола в суточной моче до 41,8 нмоль/сут. В связи с этим проведена проба с инсулиновой гипогликемией для выявления скрытой надпочечниковой недостаточности. В ходе теста достигнута истинная гипогликемия (глюкоза сыворотки крови 0,7 ммоль/л), при этом максимальный выброс кортизола составил 410,8 нмоль/л (норма более 500 нмоль/л), подтверждена вторичная надпочечниковая недостаточность. На УЗИ щитовидной железы и органов малого таза — без патологии.

Протокол МРТ головного мозга 21.04.2021.

На серии сагиттальных, корональных и аксиальных томограмм получены изображения суб- и супратенториальных структур головного мозга. В белом веществе больших полушарий, в мозолистом теле, визуализируются множественные суправентрикулярно, перивентрикулярно и юкстакортикально расположенные очаги округлой и овальной формы, с четкими контурами, размерами до 16 мм, без масс-эффекта. Инфратенториальных очаговых образований не выявлено. При контрастном усилении отмечается слабое периферическое контрастирование суправентрикулярных очагов. Срединные структуры не смещены. Желудочковая система не расширена. Субарахноидальные пространства не расширены. Определяется увеличение размеров гипофиза: вертикальный — 10 мм, поперечный — 17 мм, переднезадний — 10 мм. Верхний контур гипофиза на 2,5 мм от хиазмы. В проекции кармана Ратке имеется образование неправильной формы, гипоинтенсивное на Т2-взвешенных изображениях и гиперинтенсивное на Т1-взвешенных изображениях, размерами 8×10×9 мм, не накапливающее контрастный препарат. Ткань аденогипофиза компримирована, смещена кпереди, структура аденогипофиза однородна, при контрастном усилении аденогипофиз неоднородно накапливает контрастный препарат. Воронка не утолщена. Задняя доля гипофиза имеет типичный сигнал и локализацию, переход без особенностей.

Заключение: МР-картина демиелинизирующего заболевания с признаками обострения. Объемное образование в проекции кармана Ратке (киста кармана Ратке), с положительной динамикой по сравнению с МРТ от 29.01.2021: уменьшение размеров образования, исчезновение утолщения воронки.

Таким образом, на МРТ головного мозга через 5 мес после перенесенной коронавирусной инфекции обнаружили уменьшение размеров кисты кармана Ратке, отсутствие утолщения воронки, в целом — положительная динамика по сравнению с предыдущим исследованием (см. рис. 1). Также имеются убедительные доказательства частичного восстановления функций гипофиза после пангипопитуитаризма, развившегося в период реконвалесценции COVID-19-инфекции.

**Figure:**
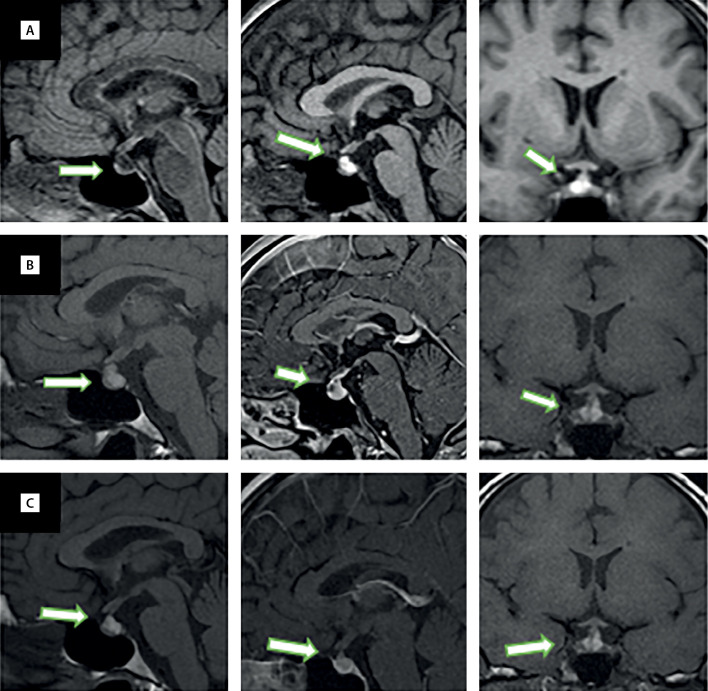
Рисунок 1. МРТ гипофиза в динамике. Т1-взвешенные изображения до введения контраста, сагиттальный срез (левая колонка), Т1-взвешенные изображения после введения контраста, сагиттальный срез (средняя колонка), Т1-взвешенные изображения после введения контраста, фронтальный срез (правая колонка). А — август 2020 г.: впервые выявленная киста кармана Ратке. В — январь 2021 г.: увеличение кисты кармана Ратке, утолщение воронки гипофиза. С — апрель 2021 г.: уменьшение кисты кармана Ратке, отсутствие утолщения воронки гипофиза.

Пациентка была выписана с рекомендациями приема гидрокортизона 5–10 мг ежедневно утром и эстроген-гестагенного препарата, содержащего эстрадиола гемигидрат 2 мг и дигидрогестерон 10 мг, 3 мес с перерывом в 2–2,5 мес и последующего динамического наблюдения.

При амбулаторном осмотре в октябре 2021 г. предъявляет жалобы на снижение либидо, набор 8 кг за 6 мес. Принимала в среднем 15 мг гидрокортизона в сутки. После отмены эстроген-гестагенного препарата в июле 2021 г. менструации отмечены в августе и октябре 2021 г. самостоятельно. При осмотре: рост 166 см, масса тела 60 кг (прибавка 8 кг за 8 мес). При обследовании уровень адренокортикотропного гормона (АКТГ) и кортизол крови утром были в норме на фоне 1,5 дневной отмены гидрокортизона (см. табл. 1), однако отмечалось снижение уровня кортизола суточной мочи до 199,41 мкг/сут при референсных значениях лаборатории 380–940 мкг/сут. Рекомендован прием гидрокортизона в дозе не более 10 мг утром. Наблюдение продолжается.

**Table:** Таблица 1. Лабораторные показатели пациентки А. в динамике

Лабораторный тест	Январь 2021	Апрель 2021	Октябрь 2021	Референсный диапазон
Кортизол сыворотки крови утром, нмоль/л	8,56	227	405	171–536
АКТГ сыворотки крови вечером/утром, пг/мл	7,68 (вечером)	33,96 (утром)	33,2 (утром)	7,2–63,3
ТТГ, мМЕ/л	1,26	2,626	2,17	0,25–3,5
Т4св, пмоль/л	6,0	10,75	11,5	9–19
ЛГ, Ед/л	0,95	4,73	-	2,6–12,1
ФСГ, Ед/л	1,86	4,52	-	1,9–11,7
Эстрадиол, пмоль/л	-	51,479	-	97–592
Пролактин общий, мЕд/л	2501	68,3	-	66–436
ИФР-1, нг/мл	95,2	210,7	-	109–284
Натрий, ммоль/л	138	142	-	136–145

## ОБСУЖДЕНИЕ

Гипофизит — это гетерогенная группа заболеваний, ассоциированная с воспалительными процессами селлярной и/или параселлярной области, что может приводить к дефициту функций гипофиза и /или масс-эффектам [8].

По этиологии гипофизит делят на первичный и вторичный (табл. 2).

К самому распространенному (около 68%) относят лимфоцитарный гипофизит, сопровождающийся лимфоцитарной инфильтрацией ацинусов гипофиза и интерстиция, женщины болеют в 4 раза чаще. Гранулематозный гипофизит встречается в 20% случаев, на препаратах присутствуют гранулемы, гигантские многоядерные клетки, лимфоциты. Более редкий IgG4 гипофизит может выявляться у 4% пациентов, чаще встречается у мужчин, морфологически представлен мононуклеарными клетками, IgG4-положительными плазматическими клетками, фиброзом. Такой тип гипофизита положительно отвечает на терапию глюкокортикостероидными препаратами, что является одним из диагностических критериев (табл. 3); также для подтверждения необходима биопсия, подтверждающая системность заболевания, высокий титр IgG4 и характерную МРТ-картину [8].

**Table:** Таблица 2. Этиология гипофизита

Первичный гипофизит	Вторичный гипофизит
Лимфоцитарный гипофизит	Аутоиммунные эндокринопатии	Системные аутоиммунные заболевания
Гранулематозный гипофизит	Аутоиммунные полигландулярные синдромы	Синдром Шегрена
IgG-4-ассоциированный гипофизит	Тиреоидит Хашимото	Системная красная волчанка
Ксантоматозный гипофизит	Болезнь Грейвса	Болезнь Бехчета
Некротизирующий гипофизит	Сахарный диабет 1 типа	Первичный билиарный цирроз
Смешанные формы (лимфогранулематозный, ксантогранулематозный гипофизит)	Болезнь Аддисона	Неврит зрительных нервов
		Атрофический гастрит
Миокардит
Болезнь КронаИдиопатическая тромбоцитопеническая пурпура
Аутоиммунный гепатит

**Table:** Таблица 3. Диагностика гипофизита

Предположительная этиология	Исследования
Лимфоцитарный гипофизит	АТ ТПО.Антинуклеарные антитела.Антитела к цитоплазматическому антигену anti-La, антитела к белку, связанному с РНК-полимеразой-3, антитела к двуспиральной ДНК, антитела к гипофизу
Гранулематозные повреждения	Рентгенография грудной клетки, измерение интерферона гамма при наличии в анамнезе путешествий или туберкулеза.Сывороточный АПФ при подозрении на саркоидоз.АНЦА-антитела.Анализ цереброспинальной жидкости на глюкозу, белок, олигоклональные полосы иммуноглобулинов.КТ или сцинтиграфия
IgG4-ассоциированное заболевание	Уровни иммуноглобулинов, особенно IgG4.ПЭТ со фтор-дезоксиглюкозой для подтверждения активности заболевания
Гистиоцитоз клеток Лангерганса	Исследование скелета, сканирование всего тела с ФДГ для определения активности заболевания
Герминома	Сывороточный или цереброспинальный альфа-фетопротеин и ХГЧ
Другая инфильтративная/инфекционная этиология	Лактатдегидрогеназа, клинический анализ крови, общий анализ мочи.Визуализация.Анализ цереброспинальной жидкости (цитология, олигоклональные полосы иммуноглобулинов)

Воспаление гипофиза приводит к недостаточности гормонов гипофиза и увеличению этой железы [9]. Воспалительный и инфильтративный процесс гипофиза может привести к компрессии хиазмы и дефектам полей зрения, снижению цветовой чувствительности и остроты зрения. Также описываются головная боль с/без тошноты, рвота.

Эндокринная манифестация включает в себя дефицит передней доли гипофиза и нейрогипофиза (несахарный диабет), а также гипо- или чаще гиперпролактинемию. Этиология гипофизита влияет на спектр развития эндокринных дисфункций. Например, при лимфоцитарном гипофизите секреция гормонов чаще снижается в следующей последовательности: АКТГ, гонадотропины, ТТГ, СТГ, однако описаны и изолированные гормональные дефициты; также чаще в острую фазу встречается гиперпролактинемия [9].

Для лабораторной диагностики гипофизита исследуют широкий спектр гормонов и некоторые биохимические показатели: кортизол, АКТГ, инсулиноподобный фактор роста-1 (ИФР-1), СТГ, эстрадиол (для женщин до менопаузы)/тестостерон, ЛГ, ФСГ, свободный Т4, ТТГ, пролактин, осмоляльность плазмы/мочи, электролиты. Рутинные базовые исследования (общеклинический анализ крови, почечный, печеночный профиль, маркеры костного обмена, С-реактивный белок, скорость оседания эритроцитов) могут пролить свет на понимание природы системного процесса.

Для визуализации гипофиза предпочтительным методом считается МРТ с контрастированием гадолинием. Изменения на МРТ, подозрительные на гипофизит, включают: быстрое, интенсивное и гомогенное усиленное накопление препарата гипофизом, без видимой патологии ножки гипофиза, у пациентов с несахарным диабетом отсутствует сигнал (свечение) от задней доли. Чаще повреждения распространяются симметрично супраселлярно, а также на прилежащую твердую оболочку, формируя «хвост твердой оболочки». Данные находки наиболее выражены у пациентов с классическим лимфоцитарным гипофизитом. Нередко присутствует «пустое турецкое седло», предположительно является результатом атрофии при затихании инфекционного процесса [9].

Прогноз для пациента определяется течением основного заболевания и степенью его компенсации, однако в большинстве случаев гормональные дефициты необратимы и прогрессируют со временем.

Целью лечения является компенсация гипопитуитаризма и ликвидация (если это возможно) воспалительного процесса в гипофизе, смягчение масс-эффекта увеличенного гипофиза.

Описываемый нами случай интересен тем, что у нашей пациентки ранее предполагался рассеянный склероз. В литературе описано всего несколько случаев сочетания гипофизита и рассеянного склероза, один из них представлен J. Pena и соавт. [10]. Авторы приводят клинический случай 13-летнего ребенка с остро развившимися признаками несахарного диабета, подтвержденного лабораторно. В день осмотра присутствовали менингизм и болезненная потеря зрения левого глаза. У ребенка были исключены аутоиммунные причины гипофизита и подтвержден лимфоцитарный генез. По данным МРТ определяли с одной стороны утолщение воронки гипофиза, с другой — очаги, характерные для рассеянного склероза на Т2-взвешенных изображениях. При лечении пульс-терапией метилпреднизолоном клиническая симптоматика уменьшилась, зрение частично восстановилось.

Однако в цитируемом случае не было зафиксировано связи с предшествующей инфекцией, что присутствовало у нашей пациентки, которая перенесла новую коронавирусную инфекцию за 2 мес до проявлений гипофизарной патологии.

Новая коронавирусная инфекция имеет множество клинических проявлений, в том числе затрагивающих эндокринную систему. К настоящему моменту еще не описаны случаи прямого воздействия COVID-19-инфекции на гипофиз или гипоталамус. Однако имеются доказательства вовлечения гипоталамо-гипофизарной оси у 61 выживших пациентов, переживших SARS-инфекцию [11]. 40% таких пациентов имели лабораторное подтверждение вторичной надпочечниковой недостаточности, которая у большинства разрешилась в течение года. Всего у 5% процентов этих пациентов также присутствовал центральный гипотиреоз. Предполагают, что в основе лежал обратимый гипофизит или повреждение вирусом гипоталамуса (прямое или иммуноопосредованное) [12][13].

Исследователи из Турции описывают клинический случай пациента 67 лет с развившимся гипопитуитаризмом через 2 мес после перенесенной коронавирусной инфекции. Заболевание манифестировало острой надпочечниковой недостаточностью, также подтвердились вторичный гипотиреоз и гипогонадизм, МРТ-картина соответствовала воспалительному процессу в области гипофиза. Состояние было компенсировано стандартной терапией [14], но указаний на обратимость эндокринных расстройств не было.

Случай нашей пациентки уникален, поскольку имеются лабораторные и инструментальные методы, подтверждающие обратимый характер пангипофизита, развившегося в периоде реконвалесценции коронавирусной инфекции, а также подтверждение того, что до инфекционного заболевания признаков гипофизита не было по данным МРТ.

Объяснить с точки зрения патогенеза такой характер течения осложнения довольно сложно. Известно, что COVID-19 взаимодействует с рецепторами ангиотензинпревращающего фермента 2 типа (АПФ2), которые экспрессируются в клетках обонятельного эпителия. Дальнейшее проникновение вируса в ЦНС не совсем понятно, однако патогенез повреждения гипофиза и гипоталамуса может быть связан с экспрессией АПФ2 в этих органах [12]. Щитовидная железа также экспрессирует рецепторы к АПФ2, которые играют ключевую роль в патологических процессах. В обсервационных исследованиях 3,6% пациентов, инфицированных COVID-19, имели какую-либо патологию щитовидной железы [15]. Прямое повреждение ткани щитовидной железы было также подтверждено на аутопсиях, а в одном из исследований заболевания щитовидной железы были ассоциированы с повышенным риском смерти у пациентов [15].

Что касается половых желез, недавнее исследование 81 мужчины, больных коронавирусной инфекцией, показало, что общий тестостерон у пациентов был ниже (хотя и статистически не значимо), а ЛГ был значительно выше по сравнению со 100 здоровыми мужчинами. Авторы говорят о необходимости аккуратной трактовки таких результатов, поскольку любое острое состояние может приводить к нарушению работы гипоталамо-гипофизарной-тестикулярной оси: снижаются уровни ЛГ, ФСГ и тестостерона [12]. Особенностью нашей пациентки является наличие у нее подтвержденного рассеянного склероза мягкого течения. Благодаря тщательному динамическому наблюдению за состоянием головного мозга мы имеем серию МРТ до заболевания COVID-19, свидетельствовавших об отсутствии патологии гипоталамо-гипофизарной области, МРТ через 3 мес после перенесенного COVID-19 с признаками гипофизита и МРТ через еще через 3 мес, подтверждающее обратное развитие патологии гипофиза.

## ЗАКЛЮЧЕНИЕ

Впервые представлен клинический случай, демонстрирующий частично обратимый пангипопитуитаризм, развившийся в период реконвалесценции новой коронавирусной инфекции у женщины репродуктивного возраста. Он демонстрирует нетипичное течение заболевания с восстановлением секреции части гормонов (ТТГ, АКТГ) и значимое клиническое улучшение в течение 3 мес с момента манифестации первых клинических признаков недостаточности гипофизарных гормонов, снижение потребности в терапии глюкокортикоидами и возможность отмены тиреоидных гормонов и нормализации уровня ИФР-1. Необходимо дальнейшее наблюдение за течением гипофизита и индивидуальный подбор заместительной терапии с учетом меняющейся потребности в гормональной терапии.

## ДОПОЛНИТЕЛЬНАЯ ИНФОРМАЦИЯ

Источники финансирования. Работа выполнена по инициативе авторов без привлечения финансирования.

Конфликт интересов. Авторы декларируют отсутствие явных и потенциальных конфликтов интересов, связанных с содержанием настоящей статьи.

Участие авторов. Горбова Н.Ю. — существенный вклад в получение, анализ данных или интерпретацию; написание статьи; Владимирова В.П. — существенный вклад в дизайн исследования и интерпретация визуализирующих методов исследования; Рожинская Л.Я. — существенный вклад в дизайн исследования, в получение, анализ данных или интерпретацию; написание статьи; Белая Ж.Е. — существенный вклад в дизайн исследования, в получение, анализ данных или интерпретацию; написание статьи. Все авторы одобрили финальную версию статьи перед публикацией, выразили согласие нести ответственность за все аспекты работы, подразумевающую надлежащее изучение и решение вопросов, связанных с точностью или добросовестностью любой части работы.

Согласие пациента. Пациент добровольно подписал информированное согласие на публикацию персональной медицинской информации в обезличенной форме.
